# Modeling mosquito-borne and sexual transmission of Zika virus in an enzootic host, the African green monkey

**DOI:** 10.1371/journal.pntd.0008107

**Published:** 2020-06-22

**Authors:** Andrew D. Haddow, Unai Perez-Sautu, Michael R. Wiley, Lynn J. Miller, Adrienne E. Kimmel, Lucia M. Principe, Suzanne E. Wollen-Roberts, Joshua D. Shamblin, Stephanie M. Valdez, Lisa H. Cazares, William D. Pratt, Franco D. Rossi, Luis Lugo-Roman, Sina Bavari, Gustavo F. Palacios, Aysegul Nalca, Farooq Nasar, M. Louise M. Pitt

**Affiliations:** 1 Virology Division, United States Army Medical Research Institute of Infectious Diseases, Frederick, Maryland, United States of America; 2 Center for Genome Sciences, United States Army Medical Research Institute of Infectious Diseases, Frederick, Maryland, United States of America; 3 Veterinary Medicine Division, United States Army Medical Research Institute of Infectious Diseases, Frederick, Maryland, United States of America; 4 Molecular and Translational Sciences Division, United States Army Medical Research Institute of Infectious Diseases, Frederick, Maryland, United States of America; 5 Aerobiology Division, United States Army Medical Research Institute of Infectious Diseases, Frederick, Maryland, United States of America; Oxford University Clinical Research Unit, VIET NAM

## Abstract

Mosquito-borne and sexual transmission of Zika virus (ZIKV), a TORCH pathogen, recently initiated a series of large epidemics throughout the Tropics. Animal models are necessary to determine transmission risk and study pathogenesis, as well screen antivirals and vaccine candidates. In this study, we modeled mosquito and sexual transmission of ZIKV in the African green monkey (AGM). Following subcutaneous, intravaginal or intrarectal inoculation of AGMs with ZIKV, we determined the transmission potential and infection dynamics of the virus. AGMs inoculated by all three transmission routes exhibited viremia and viral shedding followed by strong virus neutralizing antibody responses, in the absence of clinical illness. All four of the subcutaneously inoculated AGMs became infected (mean peak viremia: 2.9 log_10_ PFU/mL, mean duration: 4.3 days) and vRNA was detected in their oral swabs, with infectious virus being detected in a subset of these specimens. Although all four of the intravaginally inoculated AGMs developed virus neutralizing antibody responses, only three had detectable viremia (mean peak viremia: 4.0 log_10_ PFU/mL, mean duration: 3.0 days). These three AGMs also had vRNA and infectious virus detected in both oral and vaginal swabs. Two of the four intrarectally inoculated AGMs became infected (mean peak viremia: 3.8 log_10_ PFU/mL, mean duration: 3.5 days). vRNA was detected in oral swabs collected from both of these infected AGMs, and infectious virus was detected in an oral swab from one of these AGMs. Notably, vRNA and infectious virus were detected in vaginal swabs collected from the infected female AGM (peak viral load: 7.5 log_10_ copies/mL, peak titer: 3.8 log_10_ PFU/mL, range of detection: 5–21 days post infection). Abnormal clinical chemistry and hematology results were detected and acute lymphadenopathy was observed in some AGMs. Infection dynamics in all three AGM ZIKV models are similar to those reported in the majority of human ZIKV infections. Our results indicate that the AGM can be used as a surrogate to model mosquito or sexual ZIKV transmission and infection. Furthermore, our results suggest that AGMs are likely involved in the enzootic maintenance and amplification cycle of ZIKV.

## Author summary

Zika virus (ZIKV) is primarily maintained in an enzootic cycle involving nonhuman primates and mosquitoes, with epizootics and epidemics occurring when the virus is introduced into naïve populations of nonhuman primates or humans, respectively. While, the primary transmission mechanism of the virus is by the bite on an infected mosquito, ZIKV can also be sexually transmitted. In an effort to develop novel animal models to study ZIKV disease, and to better understand the role of nonhuman primates as amplification and maintenance hosts of ZIKV in nature, we modeled mosquito-borne and sexual transmission of ZIKV in the enzootic host, the African green monkey (AGM). Infection dynamics and neutralizing antibody responses in all three AGM ZIKV models (subcutaneous, intravaginal and intrarectal) in the absence of clinical illness–recapitulated reported generalized human disease course. Furthermore, we detected prolonged shedding with high viral loads and infectious virus in the vaginal swabs collected from an infected female AGM inoculated intrarectally. Notably, these results support limited human clinical evidence that ZIKV transmission can occur during female-to-male vaginal sexual acts, and furthermore indicate the existence of ZIKV super-spreaders. Finally, our results indicate sexual transmission of ZIKV could occur among infected nonhuman primates (e.g. *Chlorocebus* spp.) in Africa and may serve as a secondary transmission and maintenance mechanism in the absence of mosquito-to-nonhuman primate transmission.

## Introduction

**Fig 1:**
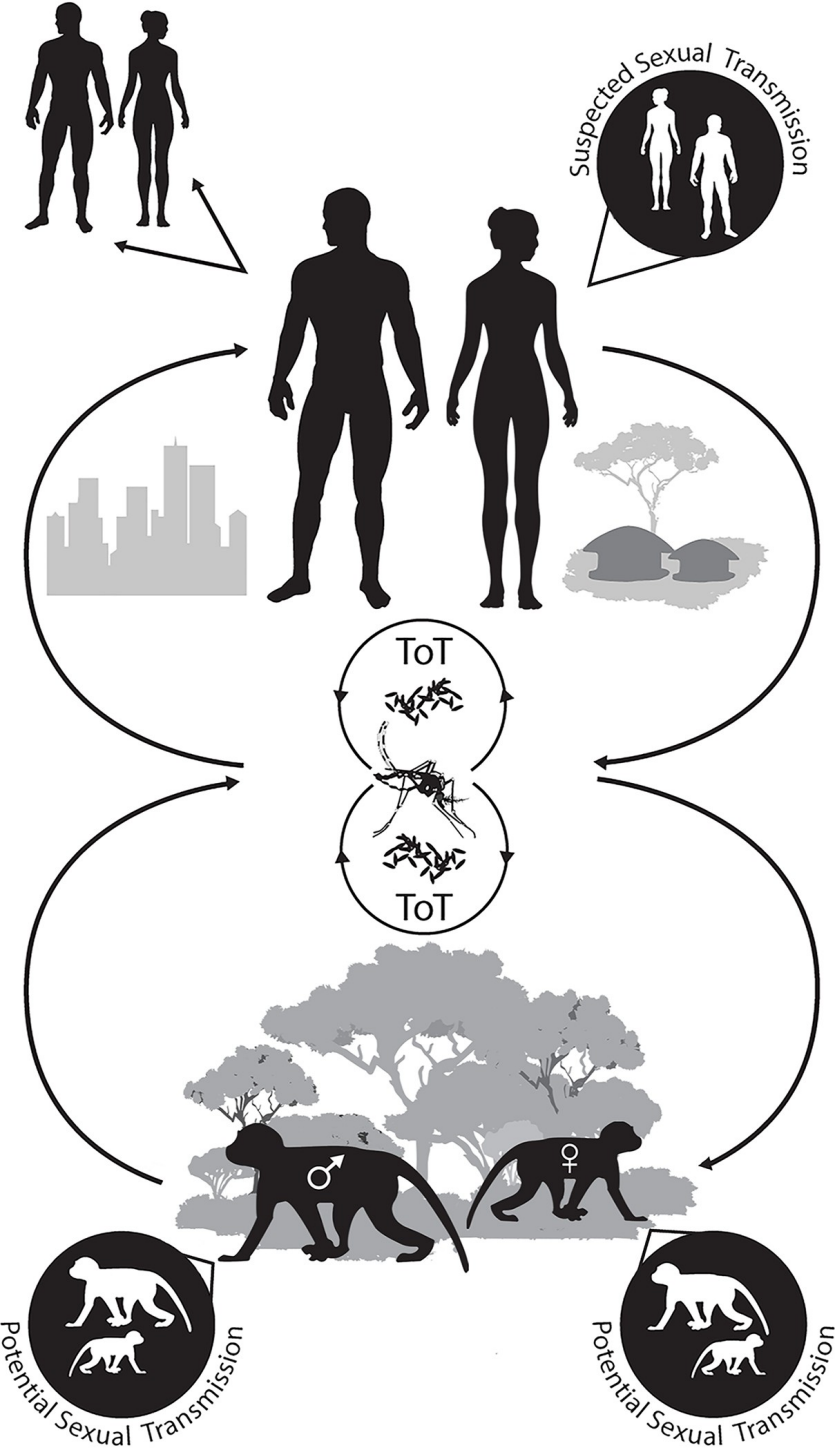
Zika virus transmission and maintenance cycles.

Zika virus (ZIKV; family *Flaviviridae*, genus *Flavivirus*), a TORCH pathogen, recently initiated a series of large epidemics in virus-naïve tropical regions that were perpetuated by mosquito-to-human and sexual transmission [1, 2] (Fig 1). The majority of human ZIKV infections are asymptomatic (≥80.0%) [1, 3–5], thus resulting case reports severely underestimate the true burden of disease. Symptomatic cases generally display a self-limiting febrile illness with common signs and symptoms including rash, fever, arthralgia, myalgia, headache, conjunctivitis, retro-orbital pain, edema, pruritus and/or fatigue [3–5]. A subset of both asymptomatic and symptomatic infections result in severe clinical manifestations including congenital birth defects (i.e. ZIKV congenital syndrome) or Guillian-Barré syndrome [1–10]. ZIKV strains comprise at least two phylogenetic lineages, African and Asian, constituting a single serotype [11–15]. While only virus strains from the Asian lineage have been reported to be teratogenic, experimental evidence is mounting that infection with strains from either lineage can result in neurological involvement following *in utero* transmission [16–18]. It is therefore possible that the lack of reported congenital birth defects in Africa may be a result of misdiagnosis, underreporting, and/or ZIKV exposure prior to puberty leading to subsequent protective immunity during a woman’s reproductive years [10]. Viremia in immunocompetent adults is generally transient, with only a fraction of cases displaying vRNA in the blood for an extended period of time [19–21]. As ZIKV has been detected and/or isolated from a variety of bodily fluids including semen, vaginal secretions and saliva [20–36], these specimen types are often used in the diagnosis of active ZIKV infection.

Although ZIKV is primarily transmitted through the bite of an infective mosquito, the virus is unique among flaviviruses, as it can also be sexually transmitted [37]. High vRNA loads (7.5–8.6 log_10_ copies/mL) have been reported in the semen of some patients [31, 36, 38–40], with virus isolations being made up to 69 days post-illness [34]. While, reported cases involving male-to-partner sexual transmission have occurred within 41 days post-illness onset [36], vRNA has been detected in the semen of previously infected males up to 9 months post-illness onset) [32, 33, 36]. Hence, prolonged viral shedding in the semen of previously infected males’ points to the potential for long-duration super-shedders. Though the majority of sexually transmitted ZIKV cases described in case reports have involve male-to-female or male-to-male transmission [37, 40–46], ZIKV has also been isolated from vaginal secretions (≥14 days post-illness) [22, 27] and vRNA has been detected for up to 180 days post-symptom onset [47], indicating the potential existence of female long-duration super-shedders. The isolation of infectious virus in vaginal secretions coupled with epidemiological case reports suggest ZIKV could potentially be transmitted from infectious females to their sexual partners [22, 27, 48]. Thus, male-to-female, male-to-male, female-to-male or female-to-female sexual transmission could result in virus transmission following the resolution of clinical illness. Nevertheless, in regions with active virus circulation this mode of transmission is generally masked by the primary transmission mode, mosquito-to-human transmission [37, 44–46]. As such, case reports involving sexual transmission primarily involve the female or male sexual partners of males who were infected with ZIKV while traveling to regions with active virus circulation [37, 40–46].

The majority of experimental animal work to date has been carried out in immunosuppressed mice to study specific aspects of transmission or viral pathogenesis [49–54], however these models are generally lethal and do not recapitulate human infection. Thus, immunocompetent nonhuman primates (NHPs) have remained the gold standard for modeling transmission risk and studying viral pathogenesis. Uniform infection is generally achieved following subcutaneous inoculation of macaques using a ZIKV dose similar to that inoculated by a mosquito bite [55–78], whereas two studies reported non-traumatic intravaginal inoculation of macaques using a dose that recapitulates human semen viral loads resulted in considerably less transmission potential [78–80]. We previously modeled ZIKV sexual transmission risk in rhesus (*Macaca mulatta*) and cynomolgus (*Macaca fascicularis*) macaques, however only 50% of the exposed macaques of each species became infected following intravaginal inoculation [79]. Researchers elsewhere investigating intravaginal ZIKV transmission reported similar results in rhesus macaques, but used repeated exposures or medroxyprogesterone, a progesterone-based contraceptive that thins the vaginal epithelium, to initiate successful transmission and subsequent infection in some resistant macaques [80]. Thus, a highly sensitive intravaginal transmission model is needed to study acute pathogenesis. As such, there is an immediate need to investigate infection dynamics in additional NHP species.

We speculated the African green monkey (AGM: *Chlorocebus sabaeus*) would be more sensitive to infection and display a higher transmission potential than rhesus macaques, as the geographic distribution of the AGM overlaps many regions with enzootic ZIKV circulation in Africa. Furthermore, there are multiple attributes that would make the AGM a valuable alternative to the rhesus macaque for modeling ZIKV transmission and infection. Both wild and captive AGMs are widely utilized in biomedical research, as such their biology is well characterized [81, 82]. Additionally, as Vero cells are derived from AGM kidney cells [83, 84], comparisons between *in vitro* and *in vivo* studies are more appropriate than those studies utilizing discordant cell lines and NHP species. In comparison to the rhesus macaque, the AGM is smaller and easier to handle, and their weights are generally sufficient to permit blood draws over multiple days. The gestation of the AGM is similar to that of rhesus macaques (approx. 165 days) [85], making them suitable to explore *in utero* ZIKV transmission and pathogenesis. Importantly, the AGM is not an endangered species, and is more easily sourced than Indian rhesus macaques.

In this study, we model mosquito and sexual transmission of ZIKV–two principle modes of virus transmission. Herein, we report ZIKV infection dynamics and moderate-duration shedding in AGMs infected by concurrent subcutaneous, intravaginal or intrarectal inoculation; and we describe a highly sensitive non-traumatic intravaginal ZIKV transmission model. We observed viremia and shedding in bodily fluids followed by a strong virus neutralizing antibody response, in the absence of overt clinical illness–infection dynamics similar to those reported in the majority of human infections. These three AGM ZIKV models will be crucial for investigating viral pathogenesis, screening antivirals and vaccine candidates, as well as providing critical data on the role of the AGM as an enzootic amplification host.

## Methods and materials

### Ethics statement

Research was conducted under an Institutional Animal Care and Use Committee (IACUC)-approved protocol at United States Army Medical Research Institute for Infectious Diseases (USAMRIID). This protocol complied with the Animal Welfare Act, Public Health Service Policy, and other Federal statutes and regulations relating to animals and experiments involving animals. USAMRIID is accredited by the Association for Assessment and Accreditation of Laboratory Animal Care International (AAALAC) and adheres to principles stated in the Guide for the Care and Use of Laboratory Animals, National Research Council (2011).

### Animal procedures

Adult AGMs originated from the Caribbean population located on St. Kitts. All AGMs were prescreened and determined to be negative for ZIKV, Herpes B virus, Simian-T-lymphotropic virus-1, Simian immunodeficiency virus, Simian retrovirus 1/2/3 antibodies, and *Mycobacterium tuberculosis*, Salmonella spp., Campylobacter spp., Hypermucoid (HVM) *Klebsiella pneumoniae*, and *Shigella* spp. infections. AGMs were individually housed throughout the duration of this study.

### Study design

The goal of the study was to determine if AGMs were susceptible to ZIKV infection by subcutaneous, intravaginal or intrarectal inoculation. Sample size estimates were based on historic reports of ZIKV experimental infections involving NHPs [55–58], and the results of our recent intravaginal and intrarectal studies in rhesus and cynomolgus macaques [79]. Power analysis with the type I error rate set to 0.05 indicated that a group size of four individuals had an 80% probability to detect infection following subcutaneous, intravaginal or intrarectal inoculation with ZIKV. Experiments were carried out simultaneously to assess the potential for temporal variation in AGM infection dynamics. Inoculation doses were based on the anticipated natural ZIKV dose that would be inoculated via infective mosquito feeding (subcutaneous route), or through sexual transmission via infective semen (intravaginal and intrarectal route). Investigators were not blinded during the course of the study.

We utilized the sylvatic ArD 41525 strain of ZIKV (Genbank Accession: KU955591), which was isolated from a pool of *Aedes africanus* mosquitoes collected in Eastern Senegal in 1984 (passage history: AP61#1, C6/36#1, Vero #3). This isolate was kindly provided by Drs. Robert Tesh and Scott Weaver at the University of Texas Medical Branch. We selected this strain due to its low passage history, an intact N-linked glycosylation site [12, 86, 87], the results of *in vivo* and *in vitro* characterization studies [87, 88], and its ability to initiate systemic infection in rhesus and cynomolgus macaques following intravaginal or intrarectal inoculation [79]. Furthermore, this strain was isolated in a region within the known distribution of AGMs. Before study initiation, the virus challenge stock was confirmed to be pure, free of *Mycoplasma* spp. and its entire genome was sequenced [86].

Prior to the initiation of experimental work, AGMs were acclimatized to their study environment for a period of 14 days (Table 1). Each inoculation group was comprised of four AGMs. Two females and two males were anesthetized and then subcutaneously inoculated between the scapulas with 4.5 log_10_ PFU of ZIKV in 1 mL PBS. Four female AGMs were inoculated intravaginally with ZIKV as previously described [79]. Briefly, anesthetized AGMs for intravaginal inoculation were placed in dorsal recumbency with their hips elevated above their torso at a 30˚ angle. A dose of 6.3 log_10_ PFU of ZIKV in 2 mL of PBS was then administered to the vaginal canal using a lubricated size 7FR infant feeding tube (Mallinckrodt Pharmaceuticals, St. Louis, MO, USA). AGMs remained in dorsal recumbency with hip elevation for 20 minutes. An additional four AGMs (2 females and 2 males) were intrarectally inoculated with ZIKV as previously described [79]. Briefly, anesthetized AGMs for intrarectal inoculation were placed in an inverted Trendelenburg position (25˚ to 30˚ down angle) and a lubricated size 7FR infant feeding tube (Mallinckrodt Pharmaceuticals) was inserted 3 to 5 cm into the rectum. A 10 mL, 0.9% sodium chloride flush was administered to soften impacted fecal material lining the rectum, after which 6.5 log_10_ PFU of ZIKV in 3 mL of PBS was administered. AGMs stayed in dorsal recumbency with hip elevation for 20 minutes.

**Table 1:** Timeline of specimens collected according to study phase.

Time	Study phase	Specimen collection schedule	Specimen type(s)*
Pre-bleed	Prior	-21 DPI	Sera
-14 to -1 DPI	Acclimatization	None	None
Pre-bleed	Prior	-7 DPI	Sera
0 DPI	Inoculation	None	None
1 to 15 DPI	Early	1–7, 9, 12 and 15 DPI	Whole blood, sera and oral swabs
1 to 15 DPI	Early	2, 5, 7, 9 and 15 DPI	Vaginal swabs
>15 DPI	Late	21 and 28 DPI	Whole blood, sera, oral and vaginal swabs

DPI, days post-inoculation.

*Sera were used for the detection of infectious virus or virus-specific neutralizing antibodies. Whole blood, oral swabs, and vaginal swabs were used for the detection of vRNA. vRNA positive oral and vaginal swabs were screened for infectious virus.

### Observations and sample collections

Observations and sample collection techniques have been previously described [79]. Following inoculation, AGMs were evaluated daily for signs of illness. Physical examinations were carried at days -7, 0 to 7, 9, 12, 15, 21 and 28 days post-inoculation (DPI; Table 2). Menstruation was noted during daily observations (days 0–28), but may have occurred on additional days (e.g. light or transient events). Femoral blood and oral swabs were collected daily from 1 to 7 DPI and then on 9, 12, 15, 21 and 28 DPI; and vaginal swabs were collected at 2, 5, 7, 9, 15, 21 and 28 DPI (Table 1).

**Table 2:** Clinical observations and physical examinations.

Clinical observations	Physical examinations
Presence or absence of rash	Presence or absence of rash
Ocular evaluation	Ocular evaluation
Appearance of joints	Joint evaluation
Motor function	Oral evaluation
Presence or absence of blood and source	Presence or absence of lymphadenopathy
Presence or absence of cough	Lymph node size
Food consumption	Dehydration-skin test time
Condition of stool	Capillary refill time
Urine output	Presence or absence of blood and source
	Severity of bleeding, if present
	Presence of absence of exudate and source
	Severity of exudate, if present
	Weight
	Rectal temperature

Clinical observations were made daily from 1–28 days post-inoculation. Physical examinations were made on -7, 0–7, 9, 12, 15, 21 and 28 DPI. Lymphadenopathy was determined via manual palpation, and measured using a ruler.

Comprehensive metabolic panels were performed on serum using a Piccolo Xpress Chemistry Analyzer and Piccolo General Chemistry 13 Panel (Abbott Point of Care, Princeton, NJ, USA). Complete blood counts were performed on whole blood using a CELL-DYN 3700 system (Abbott Point of Care). Due to our sampling schedule and the weights of the AGMs, we were only able to collect a limited amount of blood at each time point. Therefore, whole blood was used to attempt detection of vRNA (RT-qPCR), and sera were used to attempt detection of viremia (plaque assay) and virus neutralizing antibodies (PRNT_80_) (Table 3). Oral and vaginal swabs were used to detect vRNA shedding (RT-qPCR), and positive swabs were screened by plaque assay for infectious virus (Table 3).

**Table 3:** Specimens and associated Zika virus assays.

Specimen	Plaque Assay	RT-qPCR	PRNT_80_
Sera	X		X
Whole blood		X	
Oral swabs*	X	X	
Vaginal swabs*	X	X	

*vRNA positive RT-qPCR specimens were screened by plaque assay.

Rectal temperatures were taken during physical examinations and M00 radio telemetry devices (DSI, Saint Paul, MN USA) were used to monitor temperatures in real-time throughout the study [79]. To generate a single data point every 30 seconds, temperature data points were averaged and then statistically filtered to remove noise and signal artifacts (Notocord-hem Evolution software platform, Version 4.3.0.47, Notocord Inc., Newark, NJ, USA).

### Infectious virus quantification

As previously described [79], virus titration was performed on confluent Vero cell (CCL-81, ATCC, Manassas, VA, USA) monolayers in six-well plates by plaque assay. Wells were infected with serial 10-fold diluted virus in media comprised of Dulbecco's Modified Eagle Medium (Corning Life Sciences, Tewksbury, MA, USA), supplemented with 50 μg/mL gentamicin (Gibco, Carlsbad, CA, USA), 1.0 mM sodium pyruvate, 1% v/v non-essential amino acids (Sigma Aldrich, St. Louis, MO, USA); media was further supplemented with 0.5 mg/mL of amphotericin B (Gibco), 100 U/ml of penicillin (Sigma), and 100 mg/ml of streptomycin (Sigma) for oral and vaginal swab specimens for infectious virus quantification. Virus or specimens were allowed to absorb for 1 hr at 37°C and cell monolayers were overlayed with 3 mL of 1% w/v Sea-Plaque agarose (Cambrex Bio Science, East Rutherford, NJ, USA) in media. Cells were incubated at 37°C (5% CO_2_) for 4–5 days and then fixed with 4% formalin (Fisher Scientific, Waltham, MA, USA) in phosphate buffered saline (PBS; Corning Life Sciences) for 24 hrs. The agarose overlay was removed and cell monolayers were fixed and stained with 2% crystal violet (Sigma Aldrich) in 70% methanol (Sigma Aldrich). Excess stain was removed under running water. Results are reported as the number of plaque forming units (PFU)/mL, with a lower limit of detection of 1.0 log_10_ PFU/mL.

### Viral RNA extraction and quantification

As previously described [79], vRNA was extracted from whole blood, saliva and vaginal specimens inactivated in TRIzol LS Reagent (Ambion) using the phenol-chloroform extraction technique. Viral RNA was then quantified using primers and a probe targeting envelope gene bases 1188–1316 [89] in a BioRad CFX96 Touch Real-Time PCR Detection System (BioRad, Hercules, CA, USA). A standard curve was generated against a synthetic oligonucleotide and genome copies were expressed as log_10_ copies (c)/mL, with a lower limit of detection of 3.0 log_10_ c/mL.

### Serology

Plaque reduction neutralization tests (PRNTs) were used to determine seroconversion in AGMs inoculated with ZIKV as previously described [79]. Serum samples were first heat-inactivated at 56°C (30 min) and subsequently serially diluted 2-fold in PBS, mixed with an equal volume of 3.3 log_10_ PFU/mL of ZIKV after which they were incubated for 1 h at 37°C (5% CO_2_). Confluent Vero cell monolayers in 6-well plates were inoculated with a serum/virus mixture in triplicate. Plates were incubated at 37°C (5% CO_2_) for 5 days and then fixed and stained as described above. PRNT_80_ titers were calculated and expressed as the reciprocal of serum dilution yielding a >80% reduction in the number of plaques. At -21 DPI, none of the AGMs had titers of 1:20 or higher, indicating these animals had not been previously infected with ZIKV. Post-exposure sera were screened on 7, 15, 21 and 28 DPI for neutralizing antibodies. We considered those AGMs with a four-fold or greater rise in titers to have seroconverted (≥1:160).

## Results

### General observations

**Fig 2:**
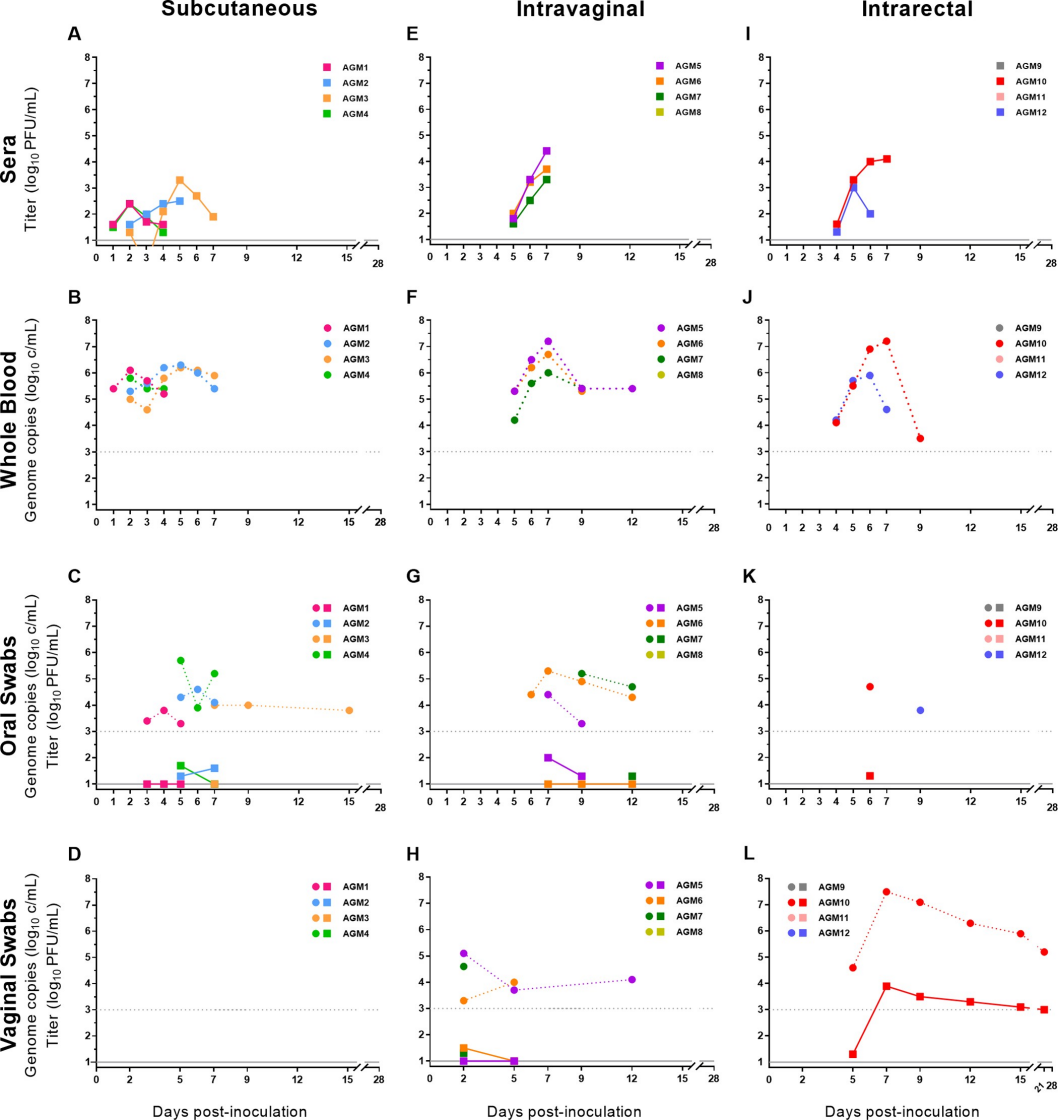
Viremia and viral RNA detected in bodily fluids by day post-inoculation. Viremia and viral RNA detected in subcutaneously inoculated African green monkeys (panel A, B, C and D); intravaginally inoculated African green monkeys (panel E, F, G and H); intrarectally inoculated African green monkeys (panel I, J, K and L). Circles and/or dotted lines connecting points represent genome copies in log_10_ c/mL. Squares and/or solid lines connecting points represent virus titers in log_10_ PFU/mL. The lower limit of detection for genome copies was 3.0 log_10_ c/mL. The lower limit of detection for virus titers was 1.0 log_10_ PFU/mL.

Throughout the course of the study, none of the infected AGMs exhibited rash, ocular abnormalities, joint swelling, decreased motor function, cough, changes in urine output, abnormal stool, decreased food consumption, pyrexia or weight loss (S1, S2, S3, S4 and S5 Figs). However, acute lymphadenopathy was observed in several AGMs (Table 4 and S6 Fig). While the study lacked the statistical power to resolve variations in laboratory values, we observed abnormal values indicative of infection in some AGMs (Table 4; S7, S8 and S9 Figs). Viremia, as measured by plaque assay, was seen in all infected AGMs with the exception of a single female AGM who seroconverted following intravaginal inoculation (Table 5). Viral shedding was observed in the saliva of all AGMs who had detectable vRNA in the whole blood (Fig 2 and Table 6), and virus was isolated in a subset of these vRNA positive specimens (Fig 2). In addition, prolonged viral shedding with high viral loads/infectious virus were detected/isolated in the vaginal swabs of a female AGM inoculated intrarectally (Fig 2 and Table 6). With the exception of the female AGM who seroconverted in the absence of detectable viremia or vRNA, all infected AGMs displayed robust virus neutralizing antibody titers (Table 5). We observed a 1:1 ratio of male to female AGMs infected by the subcutaneous or intrarectal routes, indicating a similar susceptibility to ZIKV infection for both male and female AGMs.

**Table 4:** Abnormal laboratory results and clinical observations, days 1 to 15 post-inoculation.

Animal	Route	Sex	Red blood cell parameters*	White blood cell parameters	Glucose^†^	Electrolytes*	Liver function^†^	Kidney function^†^	Pancreatic function^†^	Acute lymphadenopathy
AGM 1	SC	F	RBC, HBC, HCT	%EOS (high)	–	–	ALP, AST	–	–	Axillary
AGM 2	SC	F	RBC, HBC, HCT	%LYMPH (high)	–	–	AST, ALT	–	–	Axillary
AGM 3	SC	M	–	–	–	CA	AST, ALT, TBIL	–	–	Axillary
AGM 4	SC	M	–	–	–	–	AST, ALT, TBIL	CRE	–	–
AGM 5	IVAG	F	–	%MONO (high)	GLU	CA	AST	–	–	Inguinal
AGM 6	IVAG	F	–	WBC (high)		CA	AST	–	–	Axillary, Inguinal
AGM 7	IVAG	F	–	%NEUT (low), %LYMPH (high)	GLU		AST	–	AMY	Inguinal
AGM 8	IVAG	F	RBC, HBC, HCT		GLU	CA	AST, ALT	–	–	–
AGM 9	IR	F	RBC, HBC, HCT	WBC (high), %LYMPH (high)	GLU	CA	ALP, AST, ALT	–	–	–
AGM 10	IR	F	RBC, HBC, HCT	–	–	–	ALP, AST, ALT	BUN	–	–
AGM 11	IR	M	–	–	–	–	ALP, AST, TBIL	–	–	–
AGM 12	IR	M	–	–	–	CA	ALP, AST, ALT, TBIL	CRE	–	–

*Low.

^†^Elevated.

Red blood cell parameters: RBC (Red blood cell count), HGB (Hemoglobin), HCT (Hematocrit)

White blood cell parameters: WBC (White blood cell count), %NEUT (% Neutrophils), %LYMP (% Lympocytes), %MONO (% Monocytes), %BASO (% Basophils), %EOS (% Eosinophils)

Glucose: GLU (Glucose)

Electrolytes: CA (Calcium)

Liver function: ALP (Alkaline phosphatase), ALT (Alanine aminotransferase), AST (Aspartate aminotransferase), TBIL (Total bilirubin), GGT (Gamma glutamyltransferase)

Kidney function: BUN (Blood urea nitrogen), CRE (Creatinine)

Pancreatic function: AMY (Amylase)

No animals exhibited abnormal platelet or protein values

**Table 5:** Virus neutralizing antibody titers in African green monkeys according to route of Zika virus inoculation and sex.

Animal	Route	Sex	Serological response^†^ (PRNT_80_) DPI			
			7	15	21	28
AGM 1	SC	F	–	1:640	1:1280	1:1280
AGM 2	SC	F	–	1:1280	1:2560	1:2560
AGM 3	SC	M	–	1:640	1:640	1:1280
AGM 4	SC	M	–	1:640	1:640	1:640
AGM 5	IVAG	F	–	1:320	1:1280	1:1280
AGM 6	IVAG	F	–	1:80	1:640	1:1280
AGM 7	IVAG	F	–	1:40	1:320	1:1280
AGM 8	IVAG	F	–	1:40	1:80	1:160
AGM 9	IR	F	–	–	–	–
AGM 10	IR	F	–	1:80	1:1280	1:1280
AGM 11	IR	M	–	–	–	–
AGM 12	IR	M	–	1:320	1:1280	1:1280

DPI, Day post-inoculation. Route: SC, subcutaneous; IVAG, intravaginal; IR, intrarectal.

–<1:20. ^*^ Limit of detection 1.0 log_10_ PFU/mL.

^†^ Limit of detection 1:20.

**Table 6:** Detection of viremia, viral RNA and virus-specific neutralizing antibodies in Zika virus-infected African green monkeys.

		Number infected according to sample type/total number infected (Percent)				
Inoculation group	Number infected/total (Percent) *	Sera (Plaque assay)	Sera (PRNT_80_)	Whole blood (RT-qPCR)	Oral swabs (RT-qPCR)	Vaginal swabs (RT-qPCR)
All animals	10/12 (83.3)	9/10 (90.0)	9/10 (90.0)	9/10 (90.0)	9/10 (90.0)	4/7 (57.1)
Subcutaneous						
Males	2/2 (100)	2/2 (100)	2/2 (100)	2/2 (100)	2/2 (100)	N/A
Females	2/2 (100)	2/2 (100)	2/2 (100)	2/2 (100)	2/2 (100)	0/2 (0.0)
Intravaginal						
Females	4/4 (100)	3/4 (75.0)	4/4 (100)	3/4 (75.0)	3/4 (75.0)	3/4 (75.0)
Intrarectal						
Males	1/2 (50.0)	1/1 (100)	1/1 (100)	1/1 (100)	1/1 (100)	N/A
Females	1/2 (50.0)	1/1 (100)	1/1 (100)	1/1 (100)	1/1 (100)	1/1 (100)

*As confirmed by titration of infectious virus by plaque assay, detection of vRNA by RT-qPCR, and/or detection of virus-specific neutralizing antibodies by PRNT_80_. N/A = Not applicable

The numerical data used to create Fig 2 is included in S1 Data.

### Subcutaneously inoculated animals

#### Viremia, viral shedding, and seroconversion

Viremia (infectious virus) was detected in all AGMs (Fig 2) ranging from 1 to 7 DPI (viremia mean duration: 4.3 d), with a mean peak titer of 2.9 log_10_ PFU/mL. vRNA was also detected in the blood of all AGMs ranging from 1 to 7 DPI (mean duration 5.0 d) (Fig 2), with a mean peak vRNA load of 6.2 log_10_ c/mL. AGM3 displayed the highest detectable viremia at 6 DPI (3.3 log_10_ PFU/mL), while AGM2 displayed the highest detectable vRNA load in the blood at 5 DPI (6.3 log_10_ c/mL). All four AGMs had vRNA detected in oral swabs (Fig 2), with a mean peak vRNA load of 5.2 log_10_ c/mL. vRNA was first detected in the oral swab of AGM1 at 3 DPI and was detected up to 15 DPI in AGM3. Infectious virus was isolated from oral swabs in AGM1 at 3 and 4 DPI (1.0 log_10_ PFU/mL both days), AGM2 at 2 and 7 DPI (1.3 log_10_ PFU/mL and 1.6 log_10_ PFU/mL, respectively), AGM3 at 7 DPI (1.0 log_10_ PFU/mL), and AGM4 at 5 and 7 DPI (1.7 log_10_ PFU/mL and 1.0 log_10_ PFU/mL, respectively). vRNA was not detected in any vaginal swab from the two female AGMs. By 15 DPI, all AGMs had seroconverted (Table 5).

#### Clinical observations and laboratory results

Acute lymphadenopathy involving the left and right axillary lymph nodes was observed in AGM1 and AGM2 at 15 DPI, and AGM3 on 12 DPI (Table 4 and S6 Fig). Potentially clinically significant marked increases or decreases in laboratory values observed in infected animals for at least two days during the study period were: alanine aminotransferase, alkaline phosphatase, aspartate aminotransferase, calcium, creatinine, eosinophils, hematocrit, hemoglobin, lymphocytes, neutrophils, red blood cell count, total bilirubin (Table 4 and S7 Fig).

### Intravaginally inoculated animals

#### Viremia, viral shedding, and seroconversion

Viremia (infectious virus) was detected in 75% of AGMs (Fig 2) between 5 and 7 DPI (viremia mean duration: 3.0 d), with a mean peak titer in the sera of 4.0 log_10_ PFU/mL. vRNA was detected in the blood of all viremic AGMs ranging from 5 to 12 DPI (mean duration 4.3 d) (Fig 2), with a mean peak vRNA load of 6.9 log_10_ c/mL. AGM5 displayed the highest detectable viremia and vRNA load in the blood at 7 DPI, reaching a titer of 4.4 log_10_ PFU/mL and a vRNA load of 7.3 log_10_ c/mL. vRNA was first detected in the oral swab of AGM6 at 6 DPI and was detectable in AGM6 and AGM7 through 12 DPI, while vRNA was detected in oral swabs of AGM5 at 7 and 9 DPI (Fig 2). The mean peak vRNA load in oral swabs was 5.2 log_10_ c/mL. Infectious virus was detected in oral swabs from AGM5 at 7 and 9 DPI (2.0 log_10_ PFU/mL and 1.3 log_10_ PFU/mL, respectively), AGM6 at 7, 9 and 12 DPI (1.0 log_10_ PFU/mL, all days), AGM7 at 12 DPI (1.3 log_10_ PFU/mL), and AGM7 at 12 DPI (1.3 log_10_ PFU/mL). vRNA was detected in the vaginal swabs of AGM5, AGM6 and AGM7 by 2 DPI (Fig 2), with a mean peak vRNA load of 4.7 log_10_ c/mL. Infectious virus was detected in vaginal swabs from AGM5 at 2 and 5 DPI (1.0 log_10_ PFU/mL, both days), AGM6 at 2 and 5 DPI (1.5 log_10_ PFU/mL and 1.0 log_10_ PFU/mL, respectively), and AGM7 at 2 DPI (1.3 log_10_ PFU/mL). Although we did not detect viremia or vRNA in AGM8, all AGMs seroconverted by 15 DPI (Table 5).

#### Clinical observations and laboratory results

Acute lymphadenopathy was observed in viremic AGMs at various times during the study period (Table 4 and S6 Fig). At 4 DPI, 75% of the AGMs (AGM5, AGM6 and AGM7) displayed lymphadenopathy involving the inguinal lymph nodes. This was a transient event in AGM5 and AGM7 lasting a single day. However, AGM6 continued to display lymphadenopathy involving the axillary or inguinal lymph nodes at multiple time points. Menstruation was observed in AGM8 during inoculation, as well as at 3 and 28 DPI; but was not observed in AGM5, AGM6 and AGM7 at inoculation or post-inoculation. Potentially clinically significant marked increases or decreases in laboratory values observed in infected animals for at least two days during the study period were: alanine aminotransferase, amylase, aspartate aminotransferase, calcium, glucose, hematocrit, hemoglobin, lymphocytes, monocytes, neutrophils, red blood cell count, and white blood cell count (Table 4 and S8 Fig).

### Intrarectally inoculated animals

#### Viremia, viral shedding, and seroconversion

Viremia (infectious virus) was detected in 50% of AGMs (Fig 2) ranging from 4 to 7 DPI (viremia mean duration: 3.5 d), with a mean peak titer of 3.8 log_10_ PFU/mL. vRNA was detected in the blood of viremic AGMs ranging from 4 to 9 DPI (mean duration 4.5 d) (Fig 2), with a mean peak vRNA load of 6.9 log_10_ c/mL. AGM10 displayed the highest detectable viremia and vRNA load in the blood at 7 DPI, reaching a titer of 4.1 log_10_ PFU/mL and a vRNA load of 7.2 log_10_ c/mL. vRNA was detected in the oral swabs from AGM10 at 6 DPI and AGM12 at 7 DPI (Fig 2), with a mean peak vRNA load of 4.5 log_10_ c/mL. Infectious virus was detected in AGM10s oral swab at 6 DPI (1.3 log_10_ PFU/mL). AGM10 had prolonged vRNA and infectious virus detected in vaginal swabs from 5 to 21 DPI (Fig 2), with a peak vRNA load of 7.5 log_10_ c/mL and a titer of 3.8 log_10_ PFU/mL at 7 DPI. By 15 DPI, all AGMs with detectable viremia seroconverted (Table 5).

#### Clinical observations and laboratory results

Menstruation was observed in AGM10 at 7, 9, 12, 15, 21 and 28 DPI. Potentially clinically significant marked increases or decreases in laboratory values observed in infected animals for at least two days during the study period were: alanine aminotransferase, alkaline phosphatase, aspartate aminotransferase, blood urea nitrogen, creatinine, calcium, hematocrit, hemoglobin, red blood cell count and total bilirubin (Table 4 and S9 Fig).

## Discussion

The speed by which ZIKV spread once being introduced into the New World is uncommon among arboviruses, which are generally maintained in transmission cycles involving arthropods and virus amplification hosts in the absence of sexual transmission. The ability of a TORCH pathogen to be transmitted by hematophagous arthropod vectors requires sensitive animal models to study transmission risk and viral pathogenesis, as well as to screen antivirals and vaccine candidates. In this study, we report ZIKV infection dynamics and viral shedding in AGMs infected via subcutaneous, intravaginal or intrarectal inoculation to model mosquito-borne and sexual transmission. Our results indicate that ZIKV infection of AGMs by these routes, strain and doses produce a mild asymptomatic infection characterized by viremia, viral shedding and a strong virus neutralizing antibody response–recapitulating generalized human disease course.

Although infection by all three routes resulted in similar viremia profiles, the onset of viremia was delayed by two to three days in intravaginally and intrarectally inoculated AGMs compared to subcutaneously inoculated AGMs. A similar delay in viremia has been previously reported in ZIKV sexual transmission experiments involving NHPs [78–80]. Of interest, we detected prolonged oral shedding and the presence of infectious virus in subcutaneously and intravaginally inoculated AGMs. While experimental evidence indicates there is less potential for transmission via infectious saliva [63], super-shedders could potentiate this mode of transmission [90, 91]. Our results are similar to the majority of human case reports, which describe a generally asymptomatic infection despite low-level viremias and the detection of vRNA shedding in bodily fluids.

Even though the clinical signs and symptoms associated with ZIKV infection are now well defined, there is little published data on laboratory values of clinical interest. Notwithstanding, a recent well-documented case series involving 18 patients reported elevated liver enzymes [92]. Similar to these findings and previous NHP studies [59, 60, 79, 93], a subset of ZIKV-infected AGMs in this study displayed elevated liver enzymes consistent with acute liver involvement that resolved following the clearance of viremia. While it is also possible repeated anesthesia may have influenced some laboratory values [94], it is important to note that human infection with Spondweni virus, the closest related virus to ZIKV [15], has been reported to result in acute liver injury in some patients [10, 95]. Although reported cases of liver involvement among ZIKV patients maybe uncommon, patients with liver disease or those with comorbidities that impact liver function should be monitored for acute liver injury.

While ZIKV-infected AGMs did not display overt signs of disease, lymphadenopathy was observed in some AGMs during physical examinations. We found that the route of inoculation coincided with external lymphatic drainage, associated with axillary (subcutaneous route) and/or inguinal (intravaginal route) lymphadenopathy. Although we did not observe acute axillary or inguinal lymphadenopathy in AGMs infected via the intrarectal route, we speculate that infection could result in intra-abdominal lymph node changes. However, abdominal palpation would likely be insufficiently sensitive to detect minor-to-moderately enlarged lymph nodes in the abdomen and may require the use of ultrasound imaging. To our knowledge lymphadenopathy has not been previously reported in ZIKV-experimentally infected NHPs, though several studies reported vRNA persistence in lymphoid tissues [62, 65, 76, 78, 80, 96, 97].

Sexual transmission likely accounts for a substantial number of asymptomatic ZIKV cases [44–46], however recent NHP research has primarily focused on modeling mosquito and *in utero* ZIKV transmission rather than investigating sexual transmission risk and its resulting viral pathogenesis [14, 59, 61, 64, 65, 70–75, 98]. Previous work in macaques demonstrated high rates of infection following intravaginal or intrarectal inoculation [78–80]. In this study, we demonstrated that AGMs are highly sensitive to ZIKV infection following intravaginal inoculation. Infection with ZIKV through vaginal secretions has been identified as a possible transmission mode, and there is at least one well documented case of suspected female-to-male transmission [48]. In this study, a female AGM infected intrarectally displayed high vRNA loads and prolonged shedding of infectious virus in vaginal secretions, supporting clinical and epidemiological evidence of transmission to a sexual partner via this route. While the rate of intrarectal transmission was not as high as that observed in the intravaginally inoculated AGMs, it is important to remember that inoculation was by a one-time, non-traumatic inoculation event; thus repeated exposures, rectal trauma resulting in micro-tears during intercourse and/or the presence of co-infections could increase the chances of viral transmission [79].

The susceptibility of AGMs via all three-transmission routes has implications for zoonotic virus amplification and maintenance. The geographical range of *Chlorocebus* spp. overlaps with multiple sylvatic ZIKV mosquito vectors [1, 12, 81, 99]. Furthermore, *Chlorocebus* spp., including the AGM, are known to inhabit locations with reported ZIKV epizootics [56, 99–103]. The observed viremias among all infected AGMs in this study were sustained and likely reached titers needed to infect a portion of the principle sylvatic mosquito vectors [77]. In addition to mosquito transmission, the sensitivity to infection via intravaginal or intrarectal routes indicates the potential for sexual transmission between AGMs. Mating behaviors within AGM populations could exacerbate this potential as dominant males mate with the majority of females in a group and have multiple mating encounters with each female during the mating season [104]. Viral shedding in the vaginal secretions of female AGMs would also increase the chance of mucosal transmission during grooming, male-to-female mating, female-to-female genital investigation or female-to-female rubbing–all of which have been observed in *Chlorocebus* spp. in the wild [105]. Male-to-male mounting or anal penetration has been reported among various NHP species [105, 106], and may serve as another potential transmission mode.

Our results and recent work demonstrating ZIKV sexual transmission in macaques [79, 80], support the possibility that sexual transmission may be more common than previously suggested among various NHP species involved in ZIKV enzootic cycles. Furthermore, recent work in macaques demonstrated the potential for oropharyngeal ZIKV transmission [63]. It is therefore possible that in nature, grooming or biting by infectious AGMs shedding virus in the saliva may serve as a tertiary transmission mode, as reported in SIV transmission between AGMs [107, 108]. Super-spreaders [109, 110], could further potentiate ZIKV transmission through grooming, biting or sexual transmission. Moreover, dispersion by infected males or troop-to-troop contact could consequently initiate epizootics in virus-naïve areas or groups. Such a cycle, coupled with transovarial transmission [99, 111] and the movement of infected mosquitoes in air currents above canopies [112–116], may partially explain the long periods of increased ZIKV enzootic activity reported in Uganda and in Senegal [99, 102]. Furthermore, the wide range of ecological niches *Chlorocebus* spp. inhabit, coupled with their broad geographic distribution in Africa would increase the likelihood of virus spillover events into human populations involving terrestrial amplification hosts/mosquito vector species.

Our study has some limitations. This was a pilot study designed to investigate the susceptibility of AGMs to ZIKV infection following subcutaneous, intravaginal or intrarectal inoculation; consequently, the study was not designed with the statistical power to perform statistical analyses of chemistry, hematology or temperature data. Nevertheless, we were able to infer ZIKV-associated transient hepatic involvement based on elevated transaminase levels, similar to studies reported elsewhere [59, 60, 79, 93]. While a single uninfected intrarectally inoculated AGM displayed a substantial increase in transaminases, this AGM had historically displayed liver enzyme levels (alanine aminotransferase, alkaline phosphatase and aspartate aminotransferase) at the high-end of normal or slightly above normal; and gamma-glutamyl transferase levels observed during the study were consistent with this AGMs historic values pre-exposure. Though we were able to detect acute axial and inguinal lymphadenopathy in the majority of viremic AGMs (SC and IVAG), manual abdominal examination may have failed to reveal lymphadenopathy in the abdomen of AGMs. Therefore, future studies should consider the use of ultrasound imaging, which could also be used to detect other transient pathologic changes associated with ZIKV infection. Although infection dynamics and the lack of overt clinical signs among infected AGMs are similar to those reported in the majority of human infections, it is possible that we did not observe the full spectrum of disease presentation (i.e. severe disease) due to the size of our animal cohorts.

In summary, we report the first subcutaneous, intravaginal and intrarectal models of ZIKV infection in AGMs that recapitulates infection dynamics and lymphadenopathy reported in human cases–providing a single, easily-sourced NHP species to model mosquito and sexual ZIKV transmission. These models will be critical for investigating viral pathogenesis, as well as screening antivirals and vaccine candidates. Additionally, our results indicate the AGM is an enzootic amplification host and sexual transmission between AGMs may contribute to the maintenance of ZIKV in nature.

## Supporting information

S1 Data Excel spreadsheet containing in separate sheets, the numerical data used to create Fig 2 panels 2A, 2B, 2C, 2D, 2E, 2F, 2G, 2H, 2I, 2J, 2K, and 2L. (XLSX)

S1 Fig Subcutaneously inoculated African green monkey temperatures. (TIF)

S2 Fig Intravaginally inoculated African green monkey temperatures. (TIF)

S3 Fig Intrarectally inoculated African green monkey temperatures. (TIF)

S4 Fig African green monkey rectal temperatures. (TIF)

S5 Fig African green monkey weights. (TIF)

S6 Fig African green monkeys with observed acute lymphadenopathy. (DOCX)

S7 Fig Subcutaneously inoculated African green monkey laboratory values. (TIF)

S8 Fig Intravaginally inoculated African green monkey laboratory values. (TIF)

S9 Fig Intrarectally inoculated African green monkey laboratory values. (TIF)
