# Supplementary figures and images for: Modeling mosquito-borne and sexual transmission of Zika virus in an enzootic host, the African green monkey
Source: PLoS Negl Trop Dis. 2020 Jun 22;14(6):e0008107. doi: 10.1371/journal.pntd.0008107 (PMC7343349; doi:10.1371/journal.pntd.0008107)

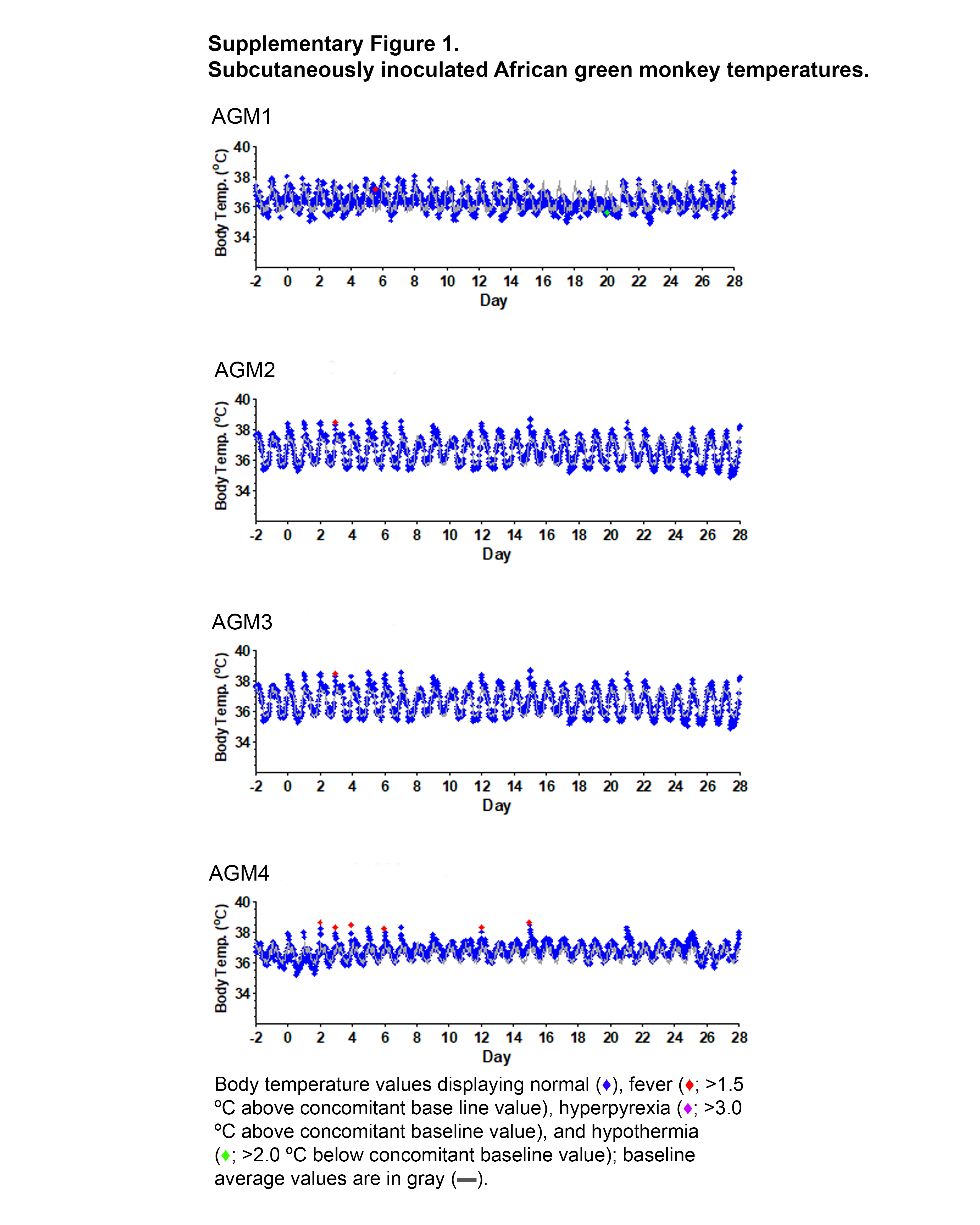

Supplement: S1 Fig — (TIF) [file pntd.0008107.s002.tif]

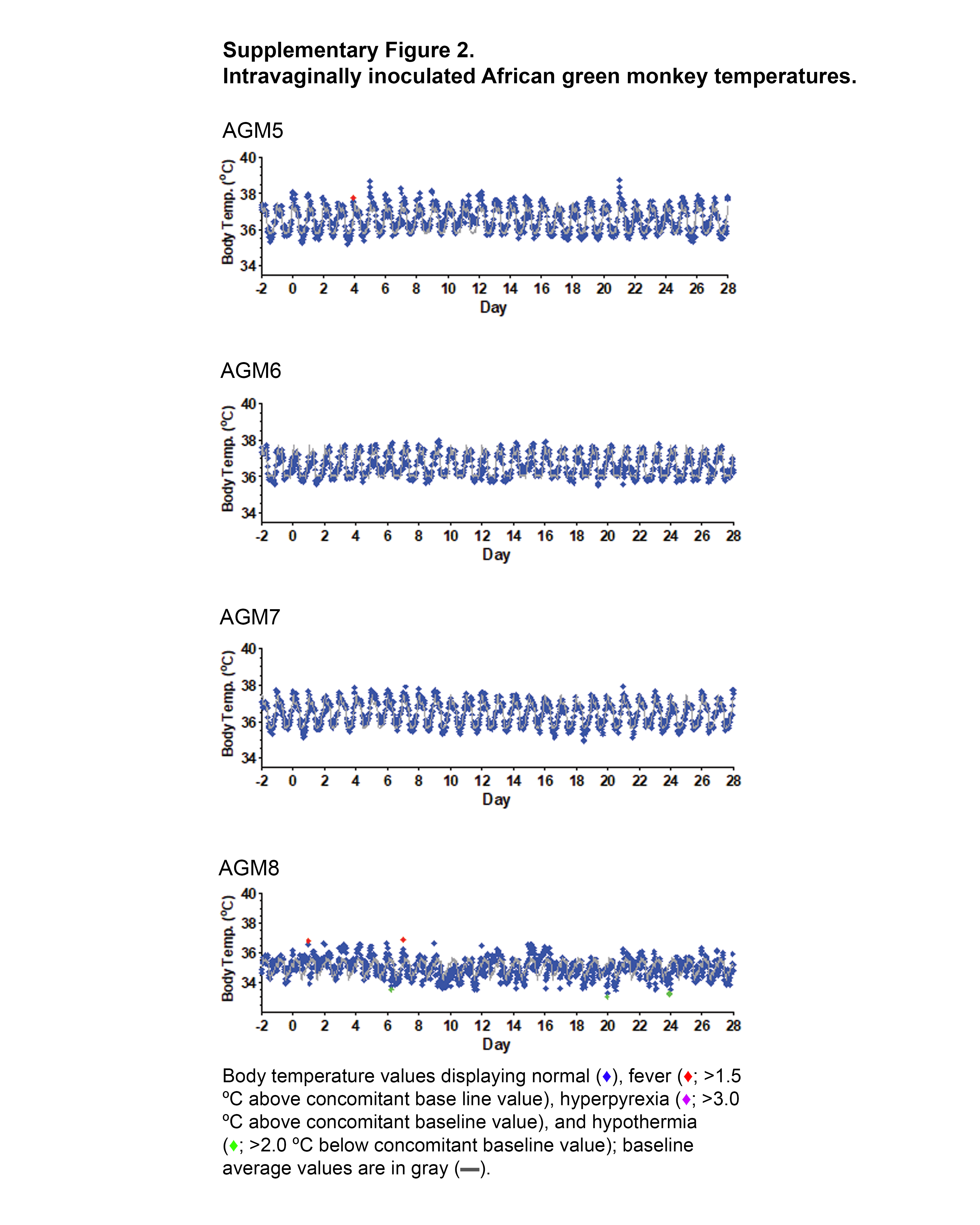

Supplement: S2 Fig — (TIF) [file pntd.0008107.s003.tif]

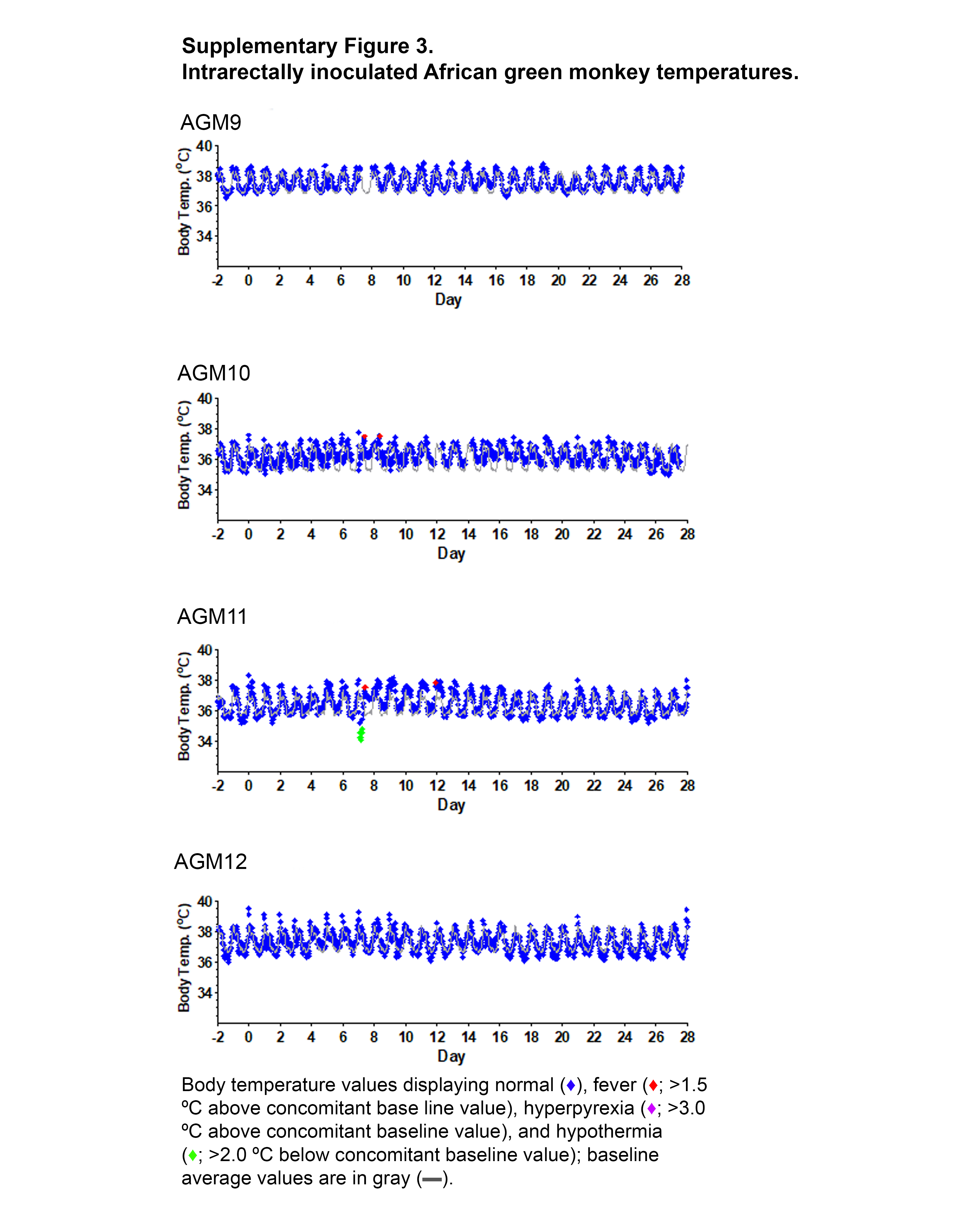

Supplement: S3 Fig — (TIF) [file pntd.0008107.s004.tif]

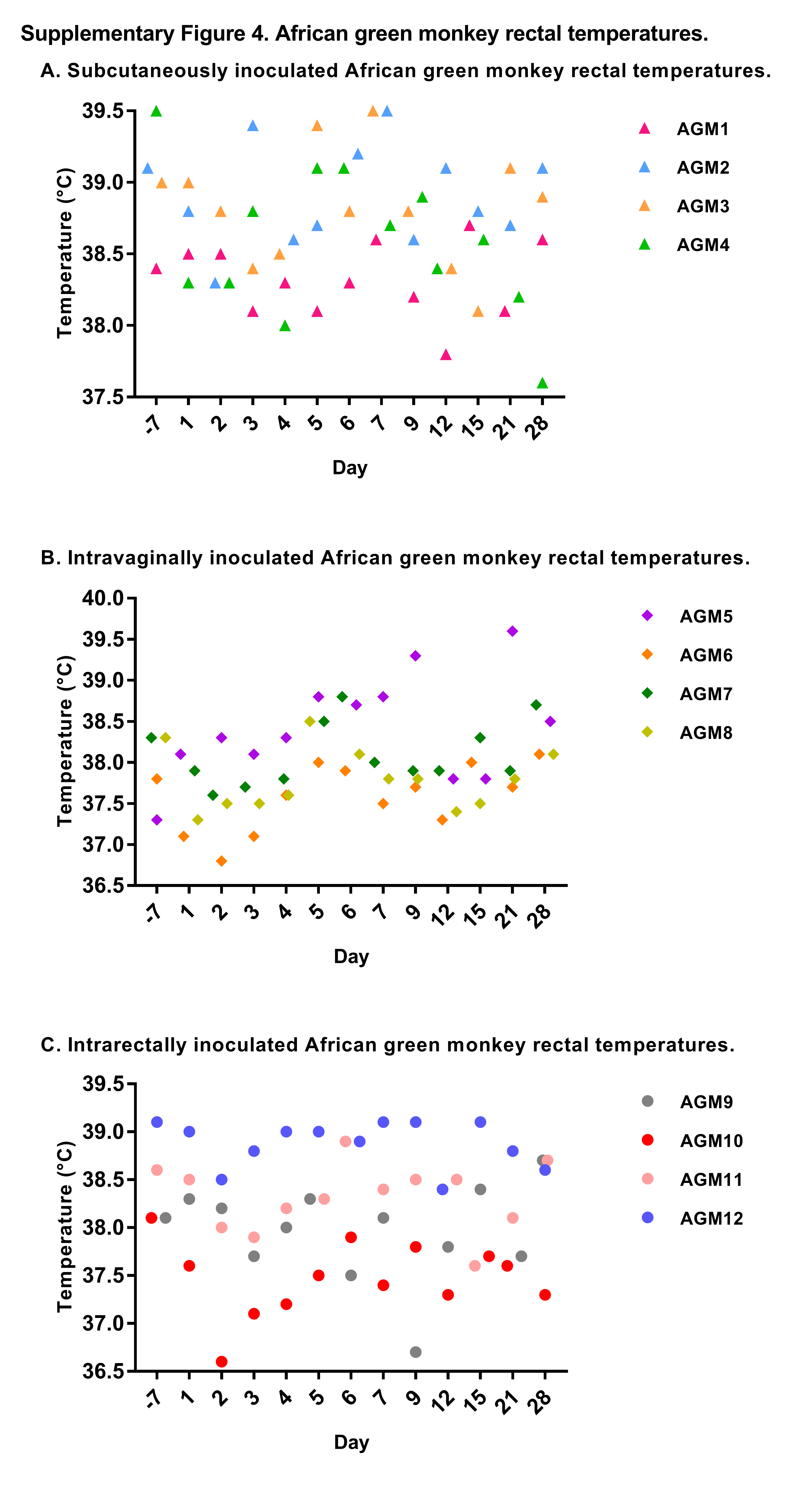

Supplement: S4 Fig — (TIF) [file pntd.0008107.s005.tif]

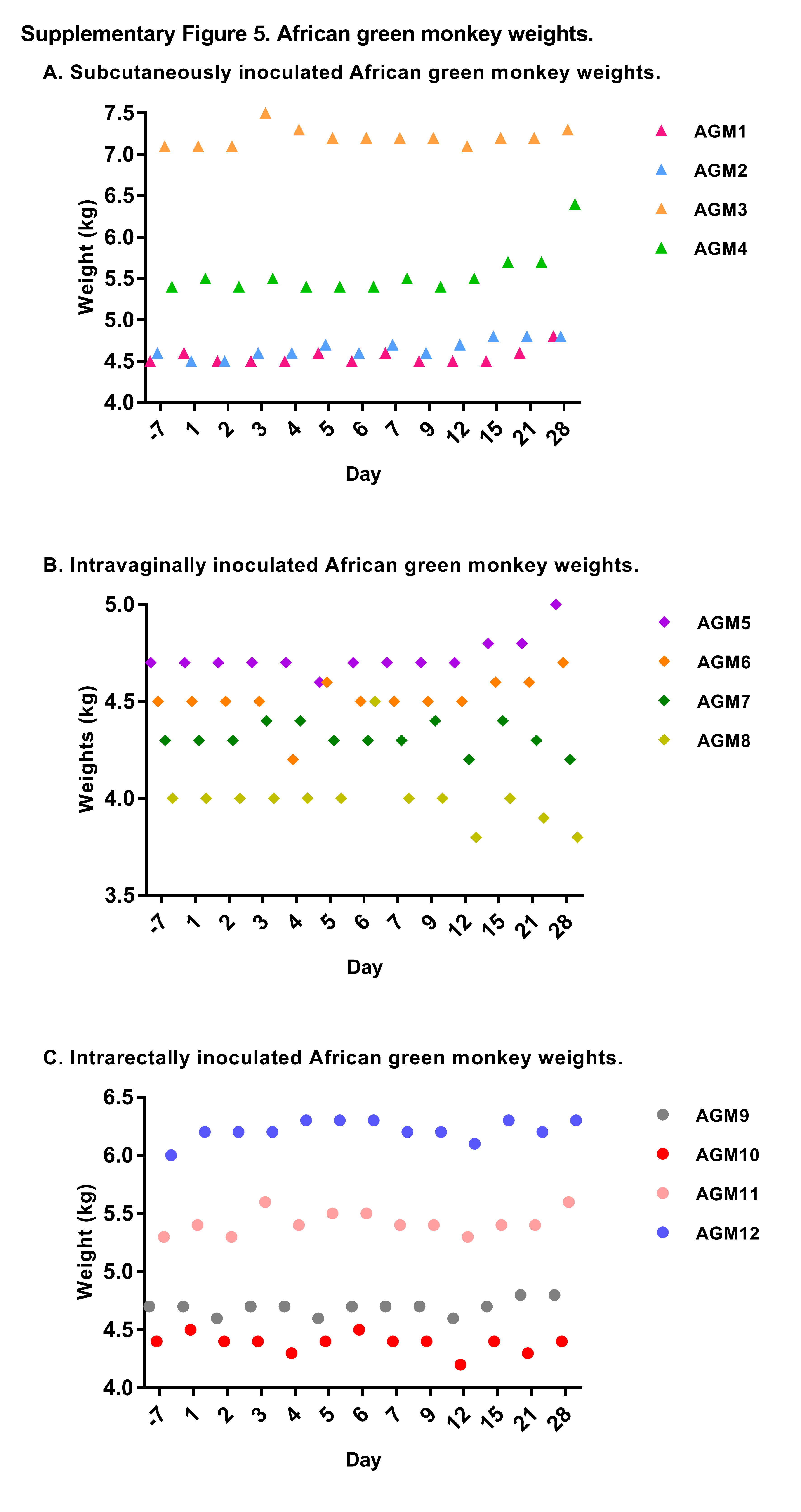

Supplement: S5 Fig — (TIF) [file pntd.0008107.s006.tif]

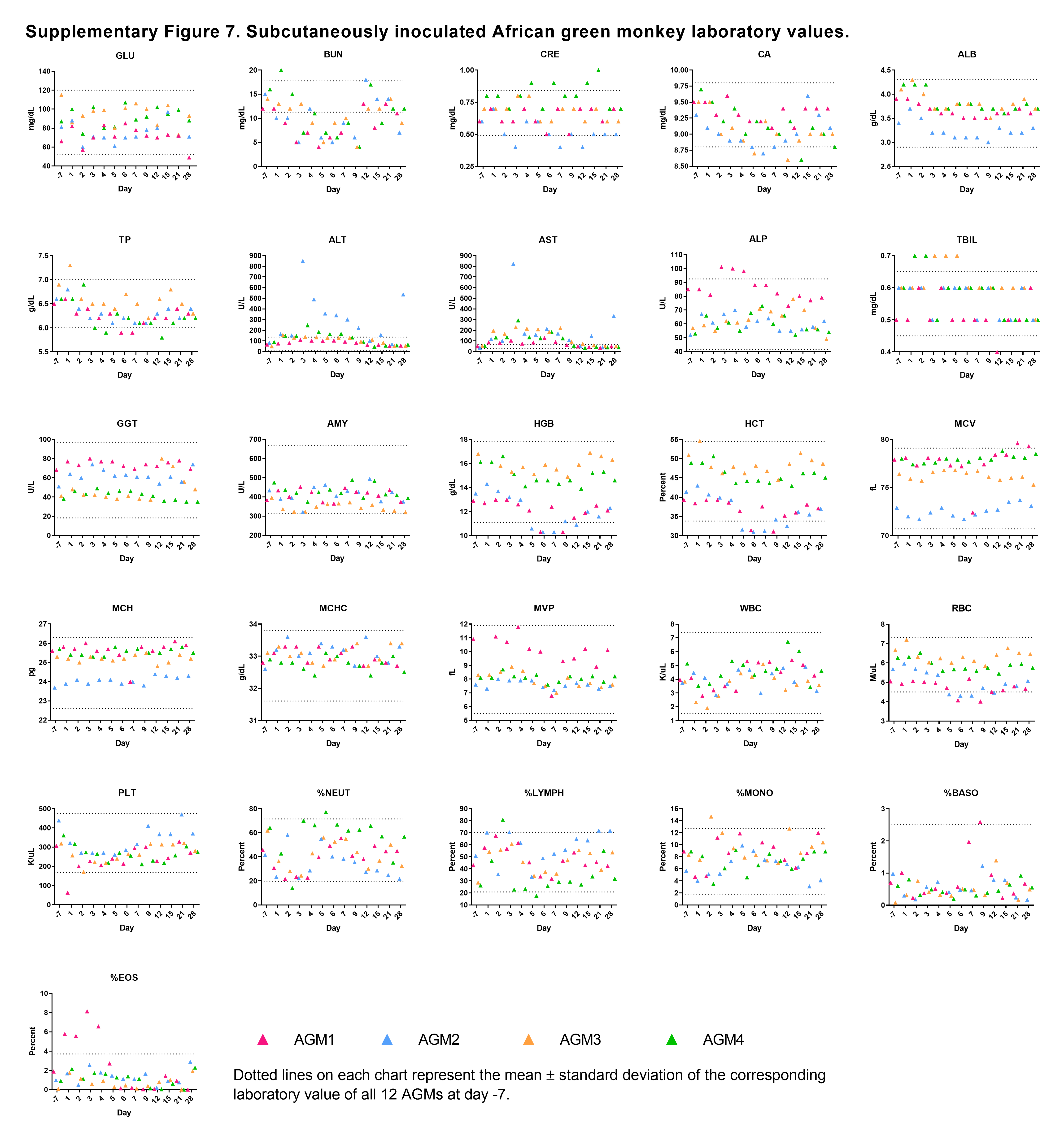

Supplement: S7 Fig — (TIF) [file pntd.0008107.s008.tif]

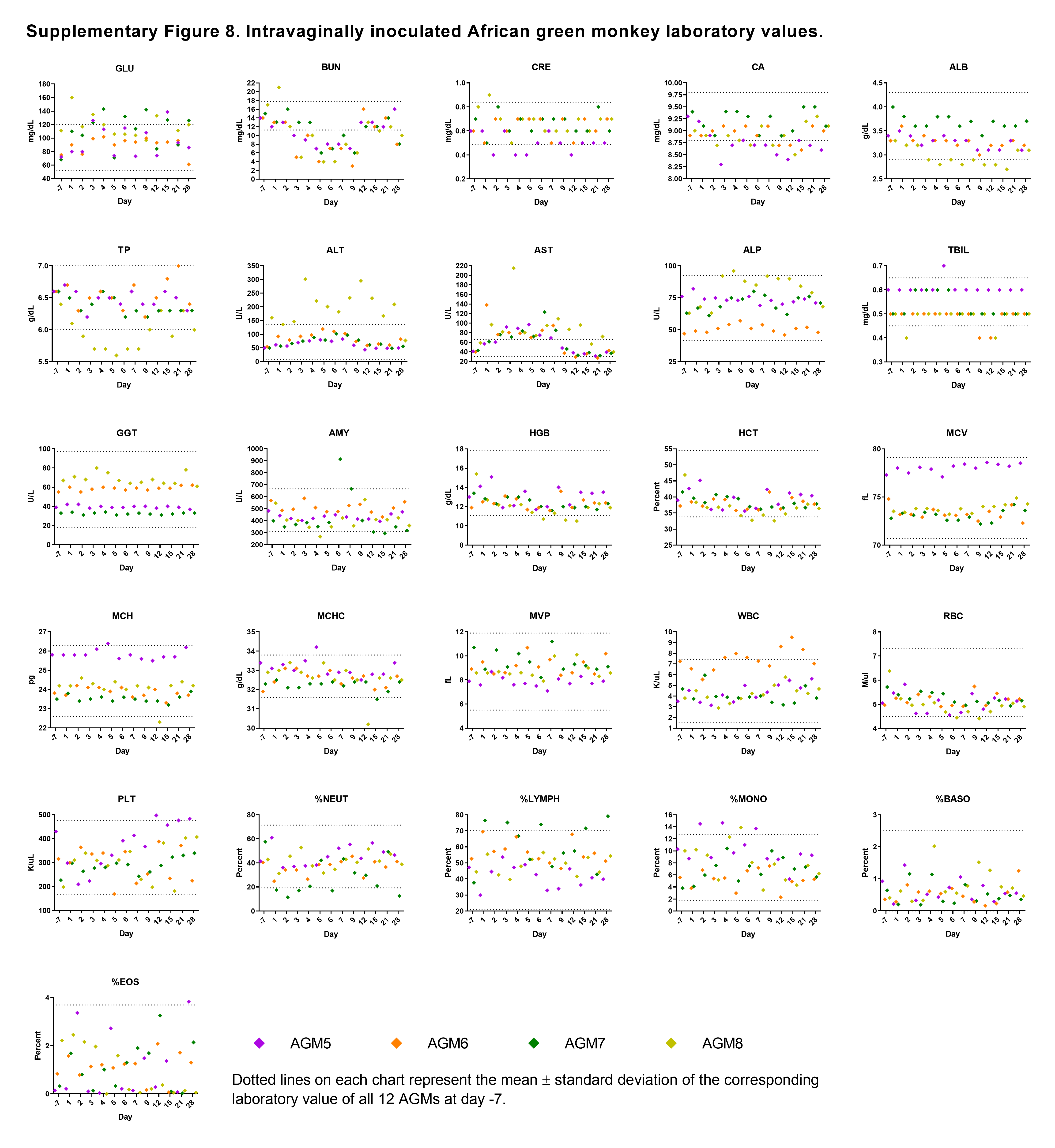

Supplement: S8 Fig — (TIF) [file pntd.0008107.s009.tif]

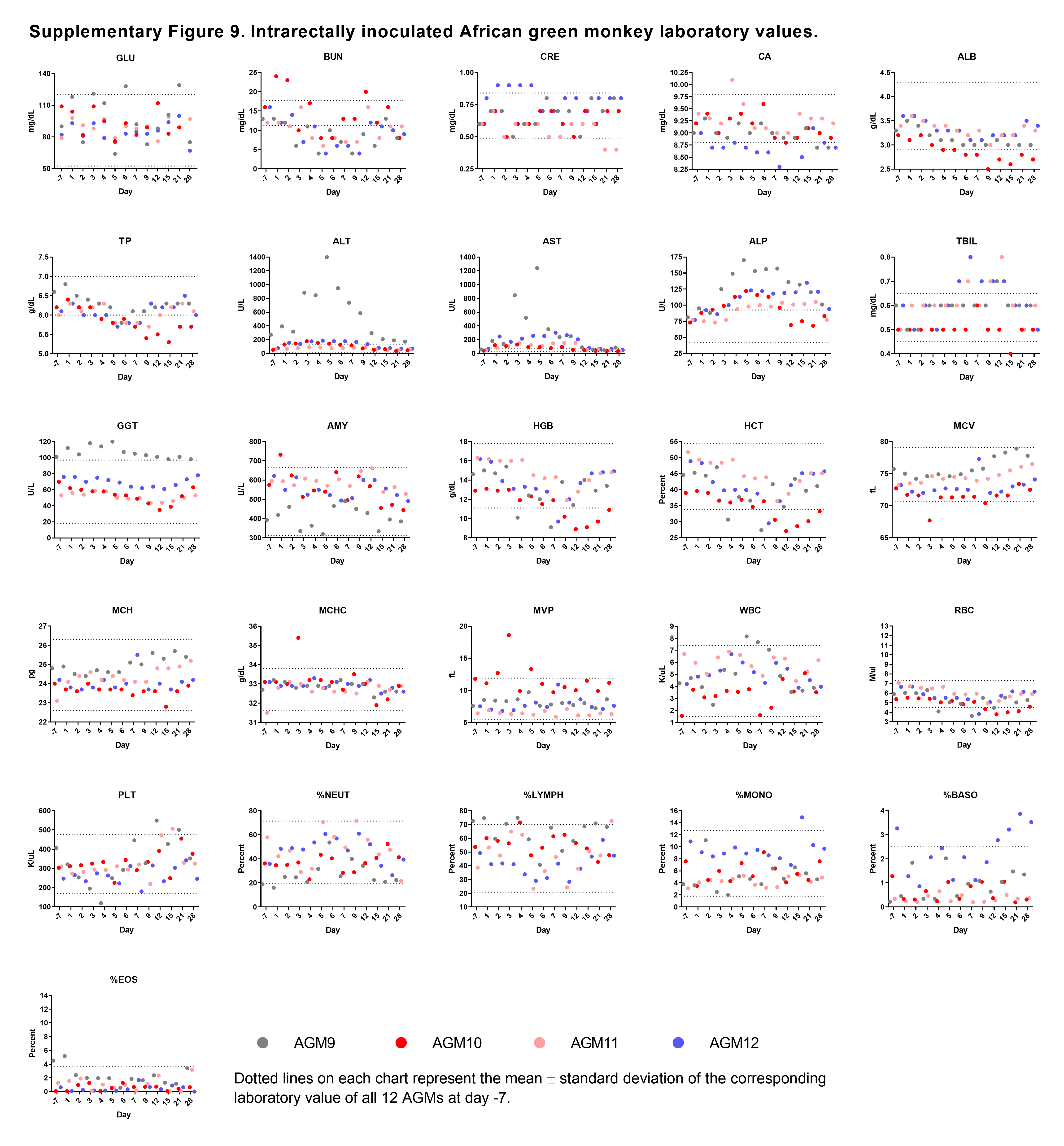

Supplement: S9 Fig — (TIF) [file pntd.0008107.s010.tif]
