# Supplementary material for: Modeling mosquito-borne and sexual transmission of Zika virus in an enzootic host, the African green monkey
Source: PLoS Negl Trop Dis. 2020 Jun 22;14(6):e0008107. doi: 10.1371/journal.pntd.0008107 (PMC7343349; doi:10.1371/journal.pntd.0008107)
Supplement: S6 Fig — (DOCX) [file pntd.0008107.s007.docx]

Supplementary Figure 6. African green monkeys with observed acute lymphadenopathy.

| **Animal and Lymph nodes** | **Sex** | **Route** | **Observed lymphadenopathy day post-inoculation** | | | | | | |
| --- | --- | --- | --- | --- | --- | --- | --- | --- | --- |
|  |  |  | **4** | **5** | **6** | **7** | **9** | **12** | **15** |
|  |  |  |  |  |  |  |  |  |  |
| **AGM1** | F | SC |  |  |  |  |  |  |  |
| Axillary |  |  |  |  |  |  |  |  | Left and Right  (5-10mm) |
| Inguinal |  |  |  |  |  |  |  |  |  |
| **AGM2** | F | SC |  |  |  |  |  |  |  |
| Axillary |  |  |  |  |  |  |  |  | Left and Right  (5-10mm) |
| Inguinal |  |  |  |  |  |  |  |  |  |
| **AGM3** | M | SC |  |  |  |  |  |  |  |
| Axillary |  |  |  |  |  |  |  | Left and Right  (5-10mm) |  |
| Inguinal |  |  |  |  |  |  |  |  |  |
| **AGM5** | F | IVAG |  |  |  |  |  |  |  |
| Axillary |  |  |  |  |  |  |  |  |  |
| Inguinal |  |  | Left  (5-10mm) |  |  |  |  |  |  |
| **AGM6** | F | IVAG |  |  |  |  |  |  |  |
| Axillary |  |  |  | Right  (5-10mm) |  |  |  | Left and Right (5-10mm) | Left and Right (5-10mm) |
| Inguinal |  |  | Left  (11-20mm) | Right  (5-10mm) |  |  | Right  (5-10mm) |  |  |
| **AGM7** | F | IVAG |  |  |  |  |  |  |  |
| Axillary |  |  |  |  |  |  |  |  |  |
| Inguinal |  |  | Left and Right  (5-10mm) |  |  |  |  |  |  |

Days 1-3 post-inoculation are not shown (no observed lymphadenopathy)
